# Delivery of Proapoptotic Agents in Glioma Cell Lines by TSPO Ligand–Dextran Nanogels

**DOI:** 10.3390/ijms19041155

**Published:** 2018-04-11

**Authors:** Antonio Lopalco, Annalisa Cutrignelli, Nunzio Denora, Mara Perrone, Rosa Maria Iacobazzi, Elisabetta Fanizza, Angela Lopedota, Nicoletta Depalo, Modesto de Candia, Massimo Franco, Valentino Laquintana

**Affiliations:** 1 Department of Pharmacy–Drug Sciences, University of Bari “Aldo Moro”, Via Orabona, St. 4, 70125 Bari, Italy; antonio.lopalco@uniba.it (A.L.); annalisa.cutrignelli@uniba.it (A.C.); nunzio.denora@uniba.it (N.D.); mara.perrone@uniba.it (M.P.); angelaassunta.lopedota@uniba.it (A.L.); modesto.decandia@uniba.it (M.d.C.); massimo.franco@uniba.it (M.F.); 2 Institute for Physical and Chemical Processes (IPCF)-CNR, SS Bari, Via Orabona, St. 4, 70125 Bari, Italy; elisabetta.fanizza@uniba.it (E.F.); n.depalo@ba.ipcf.cnr.it (N.D.); 3 Istituto tumori IRCCS “Giovanni Paolo II”, Flacco, St. 65, 70124 Bari, Italy; rosamaria.iacobazzi@gmail.com; 4 Department of Chemistry, University of Bari “Aldo Moro”, 70125 Bari, Italy

**Keywords:** translocator protein, dextran, TSPO ligand, bioconjugate, nanogels

## Abstract

Translocator protein 18-kDa (TSPO) is a versatile mitochondrial target for molecular imaging and therapy. Moreover, selective TSPO ligands have been widely investigated for diagnostic purposes and explored to target drug delivery systems directed to cancer cells overexpressing TSPO. Indeed, poly(d,l-lactic-co-glycolic acid (PLGA) polymers and nanocarriers decorated with TSPO ligands are capable of transporting TSPO ligands inside cancer cells, inducing survival inhibition in cancer cells and producing mitochondrial morphology modification. The aim of this work was to prepare nanogels (NGs) made with TSPO ligand dextran conjugates (TSPO-Dex) that are useful as potential delivery systems of two TSPO ligands as apoptotic agents. Synthesis and complete characterization of TSPO–dextran conjugates, an average molecular weights analysis, TSPO ligand release profiles, thermal behaviour and swelling studies were achieved. NG preparation, characterization and in vitro biological studies were also performed. The release of TSPO ligands released from dextran conjugates at 37 °C occurred in human serum at a faster rate than that detected in phosphate buffer. Cytotoxicity studies demonstrated that NGs produced from TSPO ligand–dextran conjugates induce survival inhibition in rat C6 glioma cell lines. Cellular uptake was also proven by fluorescence microscopy.

## 1. Introduction

Dextran is a natural and branched polysaccharide composed of linear α (1→6) linked glucose units and α (1→3) link initiated branches. Dextrans are hydrophilic and biodegradable polymers, and due to their excellent biocompatibility, are currently used in many applications, especially for biomedical purpose, such as plasma volume extenders in hypovolaemia, stabilizers, matrix components, binding platforms, lubricants and antithrombotic drugs [1]. These polysaccharides, due to their unique physicochemical properties, should be suitable as start polymers for the preparation of dextran-based bioconjugates, hydrophilic nanoparticles (NPs) and nanogels (NGs).

Translocator protein 18-kDa (TSPO) [1,2], is a mitochondrial protein that forms the trimeric complex, mitochondrial permeability transitional pore (MPTP). TSPO, formerly known as peripheral-type benzodiazepine receptor (PBR), is anatomically and pharmacologically distinct from the central-type benzodiazepine receptor (CBR) and has been recognized as a suggestive subcellular target for molecular imaging and therapy. Hence, selective TSPO ligands may be able to target drug delivery systems for the therapy of diseases overexpressing TSPO, such as tumours [3,4,5]. TSPO plays a key role in cells since is involved in several physiological processes involving mitochondria, such as steroidogenesis (e.g., cholesterol translocation into mitochondria), mitochondrial respiration, cellular immunity, and apoptosis [6]. Moreover, its overexpression, compared to healthy conditions, has been observed in the mitochondria of inflammatory cells, in activated microglia in the brain, and in several tumors [7].

**Figure 1:**
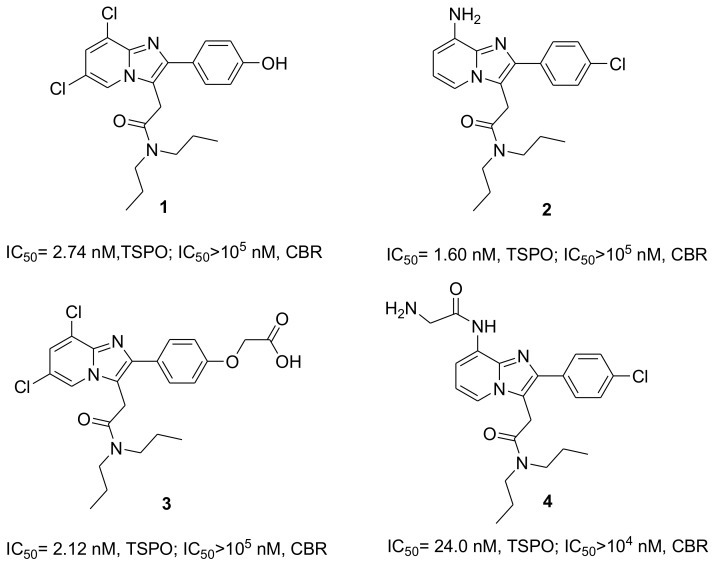
Chemical structures of selective Translocator protein 18-kDa (TSPO) ligands **1**–**4** and their affinities and selectivity vs. central-type benzodiazepine receptor (CBR).

Cholesterol and porphyrins are known as endogenous ligands for TSPO. Moreover, synthetic compounds, including phenylisoquinoline carboxamides (e.g., PK 11195), phenylbenzodiazepines (e.g., Ro-5-4864), phenoxyphenyl acetamides (e.g., DAA1106), phenoxypyridine acetamides (e.g., PBR28), imidazopyridine acetamides (e.g., Alpidem) and pyrazolopyridine acetamides (DPA713) have been largely investigated as selective TSPO ligands, for example, in binding studies, imaging and in therapeutics applications [8,9,10,11,12,13,14,15,16]. Additionally, TSPO-specific ligands (Figure 1), such as *N*,*N*,-di-*n*-propyl-[2-(6,8-dichloro-2-(4-hydroxyphenyl)imidazo[1,2-a]pyridin-3-yl)]acetamide (CB185, **1**) and [2-(4-chlorophenyl)-8-amino-imidazo[1,2-a]pyridin-3-yl]-*N*,*N*-di-*n*-propylacetamide (CB86, **2**) have been identified as agents able to induce apoptosis and cell cycle arrest in cancer cells [17]. Moreover, TSPO ligand **3** was derived from compound **1** through the addition of an acetic group and then conjugated with the fourth generation [G(4)-PAMAM] dendrimer, in order for it to target mitochondria. This was demonstrated by co-localization studies accomplished with CAT (Confocal-AFM-TIRF) microscopy [18].

TSPO ligand **4** was obtained from compound **2** by introduction of a glycine moiety to the amino group, for the synthesis of TSPO ligand bioconjugates and TSPO ligand–methotrexate prodrugs. The selective TSPO ligands **1** and **4** were conjugated with biodegradable poly(d,l-lactic-*co*-glycolic acid) (PLGA) polymers, and the macromolecular conjugates obtained showed high affinity for TSPO. Furthermore, TSPO ligand–PLGA conjugates have been shown to transport TSPO ligands inside cancer cells, induce survival inhibition in cancer cells and produce mitochondrial morphology modifications [19]. On the other hand, 5-fluorouracil drug loaded nanoparticles made from these PLGA conjugates showed a synergistic effect against C6 rat glioma cancer cells, due to the presence of TSPO ligand **1** with an anticancer drug [20]. Similarly, synergism was also highlighted for the antitumor activity of paclitaxel and methotrexate when there was simultaneous transport of TSPO ligand **2** loaded in micelles and TSPO ligand **4** as a prodrug conjugate into the cancer cells [19,21]. On the basis of the favorable results described above, and with the aim of preparing new drug release systems based on hydrophilic and biocompatible dextran, we have made new conjugates using the TSPO ligands **2** and **4** as reference models. Thus, the ultimate purpose of this work was to prepare NGs made with TSPO ligand–dextran conjugates (TSPO-Dex) that can be useful as potential delivery systems for apoptotic agents. NGs are sub-micron size nanoparticles with hydrogel properties produced by a three-dimensional polymer network. Then, NGs can be considered as promising candidates for drug delivery due to their biocompatibility, drug release and bioconjugation capacity. In addition, NGs are able to release drugs with several mechanisms, such as diffusion, swelling, or chemically-controlled process and/or in response to a wide variety of environmental stimuli [22]. Here, we have reported the synthesis and complete characterization of TSPO-Dex, in terms of average molecular weights, TSPO ligand release profiles and thermal and swelling behavior. Moreover, NG preparation, characterization and in vitro biological studies have been also performed and are reported on.

## 2. Results and Discussion

Dextran, a biodegradable and biocompatible polymer, has a number of hydroxyl groups which can be chemically modified to prepare dextran-based TSPO ligand conjugates. These compounds were processed to yield physical nanoparticles with a hydrogel network, known as NGs.

### 2.1. Synthesis and Characterization of the TSPO Ligand–Dextran Conjugates

**Figure 2:**
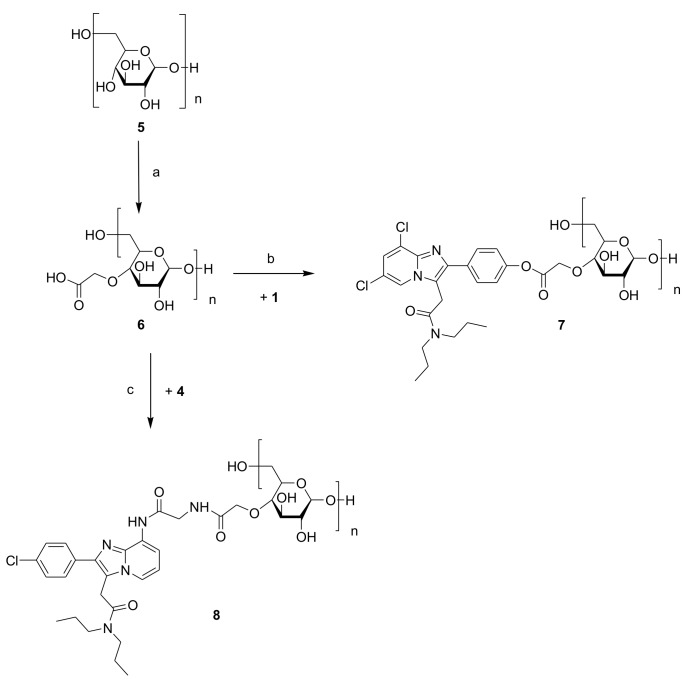
Synthesis of TSPO ligand–dextran conjugates **7** and **8**. Reagents and conditions: (a) Chloroacetic acid and NaOH 6N, r.t.; (b) EDC, HOBt, TEA and anhydrous DMF, r.t.; (c) CDI, TEA and anhydrous DMF, r.t.

**Figure 3:**
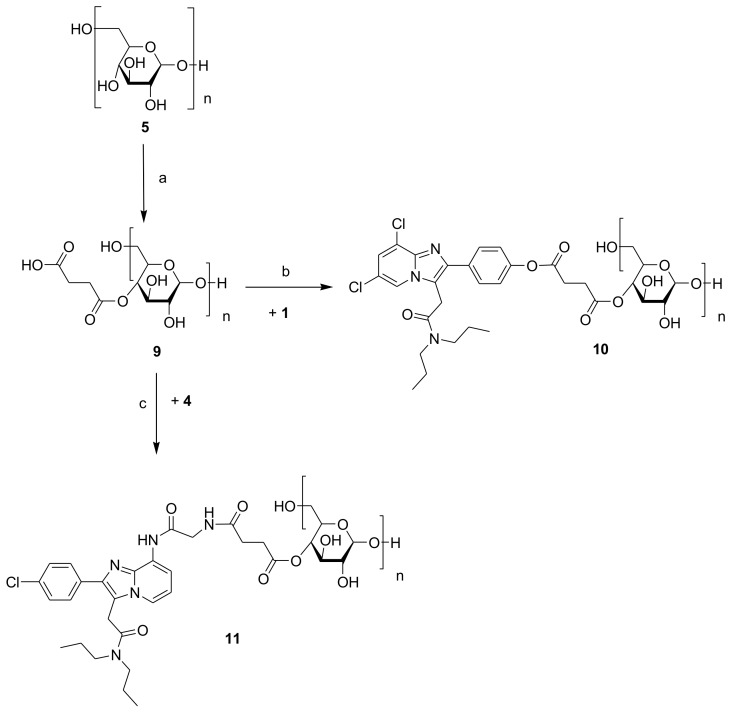
Synthesis of TSPO ligand–dextran conjugates **10** and **11**. Reagents and conditions: (a) Succinic anhydride, LiCl, anhydrous DMF and anhydrous pyridine (10% *v*/*v*), r.t.; (b) EDC, HOBt, TEA and anhydrous DMF, r.t.; (c) CDI, TEA and anhydrous DMF, r.t.

TSPO-Dex was synthetized according to the synthetic procedures reported in Figure 2 and Figure 3.

TSPO ligands **3** and **4** (Figure 1) were conjugated to carboxylated dextran derivatives (C-Dex) **6** and **9** via an ester or amide bond, which, in turn, were prepared by the reaction of dextran 40 kDa with chloroacetic acid or succinic anhydride. The reactions between C-Dex **6** or **9** with TSPO ligand **3** in anhydrous DMF at room temperature and in the presence of EDC/HOBt and TEA as coupling and deprotonating agents, respectively, yielded dextran conjugates **7** and **10**. Both form an ester bond through the reaction of 4′-phenolic group of ligand **1** with the carboxylic end groups of **6** and **9**. On the other hand, TSPO-Dex **8** and **11** were respectively synthesized by condensation in anhydrous DMF at r.t. of the carboxylic group of C-Dex **6** and **9** with the amino group of ligand **4** in the presence of a condensing agent (CDI) and a deprotonating agent (TEA) through the formation of an amide bound. Functionalization of dextran **5** with succinic anhydride was performed using pyridine as a catalyst and the DMF/LiCl system as a solvent to obtain C-Dex **9**.

**Figure 4:**
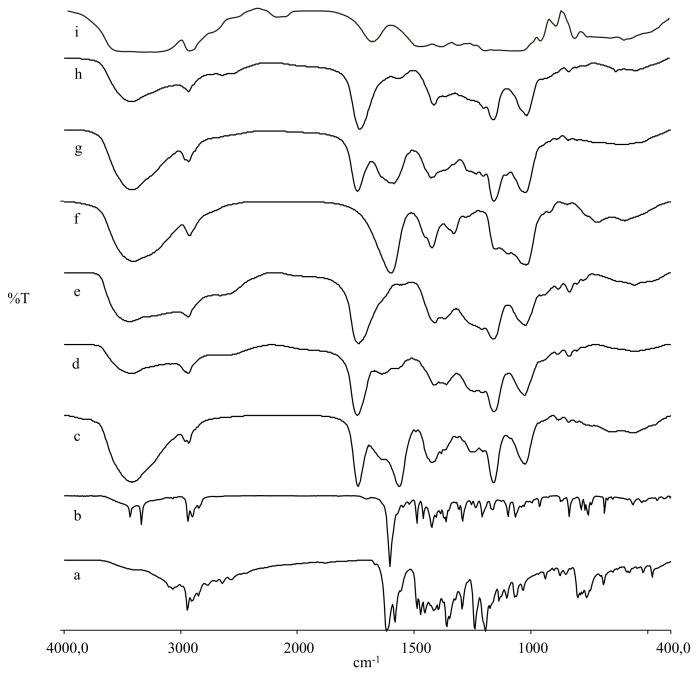
FT-IR spectra of dextran, TSPO ligands, carboxylated dextran derivatives and TSPO ligand conjugates: **1** (a), **4** (b), **11** (c), **10** (d), **9** (e), **8** (f), **7** (g), **6** (h), and **5** (i).

The purification of all conjugates was performed by exhaustive dialysis of deionized water, followed by freeze-drying of the dialyzed clear solutions. The chemical structures of C-Dex **6** and **9** and TSPO-Dex were evaluated by FT-IR, UV and ^1^HNMR spectra and elemental analysis (EA), and the molecular weight was established by Size Exclusion Chromatography (SEC) analysis. The FT-IR spectra of intermediate **6** showed the carboxyl group stretching vibration band at 1732 cm^−1^. C-Dex **9** and TSPO-Dex **7**, **10** and **11** exhibited the stretching signal of the ester band at 1736–1745 cm^−1^, partially overlapping the bands of residual carboxylic groups and amide groups, while TSPO-Dex **8** displayed the stretching signal of the amide band near 1600 cm^−1^, partially overlapping the band of residual carboxylic groups (Figure 4).

The elemental analysis of the conjugates was performed after secondary freeze-drying at 30 °C under high vacuum (<0.1 mbar) to remove the water retained by the samples as crystallization water and the amount of nitrogen obtained supports the conjugation of C-Dex with TSPO ligands (Table 1). The conjugates showed a maximum value of absorption at λ = 249–260 nm for the starting ligands in the UV spectra analysis (Table 2). The ^1^H-NMR spectra of the conjugates showed signals attributable to protons of the glucose units, together with signals of TSPO ligands. The glucose ring protons (H_2_–H_6_) signals fell between 3–4 ppm, overlapping some protons of the TSPO ligand moiety, thus, they may not be easily distinguished, while both the aromatic protons of the imidazopyridine and those of the acetamide chain have been clearly assigned. Moreover, the propensity of the conjugates to jellify in aqueous solution or DMSO complicated the separation of the signals and thus, the precise characterization of the chemical shifts that fell below the glucose ring protons. The results of ^1^H-NMR, UV and FT-IR proved the successful synthesis of TSPO-Dex. 

### 2.2. Molecular Weight by SEC Analysis

**Figure 5:**
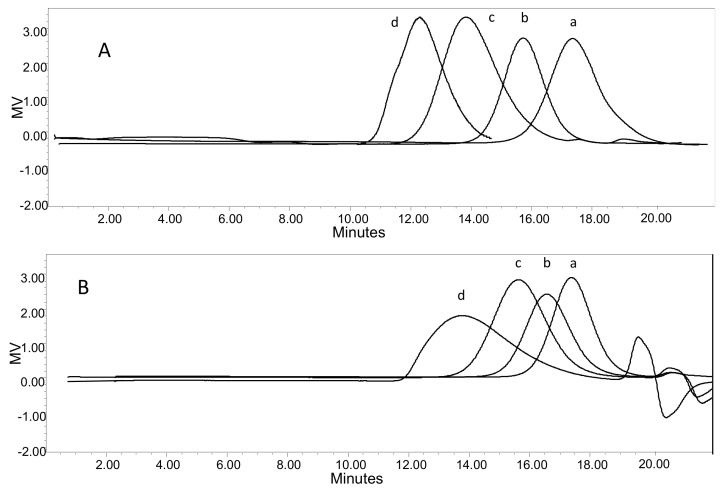
SEC elution profiles of (**A**) dextran **5** (a), carboxylated dextran derivative **6** (b), TSPO ligand conjugates **7** (c) and **10** (d); (**B**) dextran **5** (a), carboxylated dextran derivative **9** (b), TSPO ligand conjugates **8** (c) and **11** (d).

The average molecular weights and distributions of dextran **5**, C-Dex derivatives **6** and **9** and TSPO-Dex conjugated **7**, **8**, **10** and **11** were determined by SEC. The results are reported in Table 1. The average molecular weight (*M_w_*) observed for the starting dextran was 42.9 kDa and increased for derivatives and conjugates, as expected. In particular, ***M_w_*** ranged from 128.4 to 150.5 kDa for conjugates **7** and **10** and from 139.3 to 142.1 kDa for conjugates **8** and **11**, respectively. The polydispersity index (PDI) for the *M_w_*/*M_n_* of all synthesized polymers was in the range, 1.75–2.01, indicating broad dispersion of molecular weights. Figure 5 presents typical elution profiles of derivatives and conjugates.

### 2.3. Determination of Carboxylic Group Substitution Degree of the Carboxylated Dextran Derivatives and TSPO Ligands Content in TSPO Ligand–Dextran Conjugates

Acid numbers (*n*), of C-Dex **6** and **9**—measures of the carboxylic acid contents of the carboxylated dextran derivatives—were determined by acid–base titration [19]. The carboxylic group substitution degree of the C-Dex was also measured by ^1^H-NMR, whereby the integral values of the peaks at δ 4.99 ppm, assigned to CH_2_ of –O-CH_2_-COOH (compound **6**), or at 2.33 and 2.52 ppm, assigned to CH_2_ of –O-CO-(CH_2_)_2_-COOH (compound **9**), were compared with the integral of the multiplets at δ 3.2–4.0 ppm of glucose ring protons (H_2_–H_6_). The pH analysis data and ^1^H-NMR results were consistent with each other. The conjugation degree of the TSPO-Dex was estimated by UV spectroscopy by comparing TSPO-Dex E^1%^ values with those of pure TSPO ligands, measured at their absorption maximum wavelength (λ = 254 nm). The conjugation degree was also calculated by elemental and SEC analyses, using the percentages of nitrogen detected and molecular weights, respectively, and both results were in good agreement with those obtained by UV analysis. The number of residual free carboxylic groups of TSPO-Dex were calculated by the difference between the number of moles in the conjugated ligand and of the number of carboxylic groups in the C-Dex intermediate compounds. These results are reported in Table 2. TSPO-Dex conjugates showed a conjugation degree of 0.25 to 0.37 mol/mol, that corresponded to a ligand content by weight between 30% and 60% per gram of polymer.

### 2.4. Differential Scanning Calorimetry (DSC) Studies

**Figure 6:**
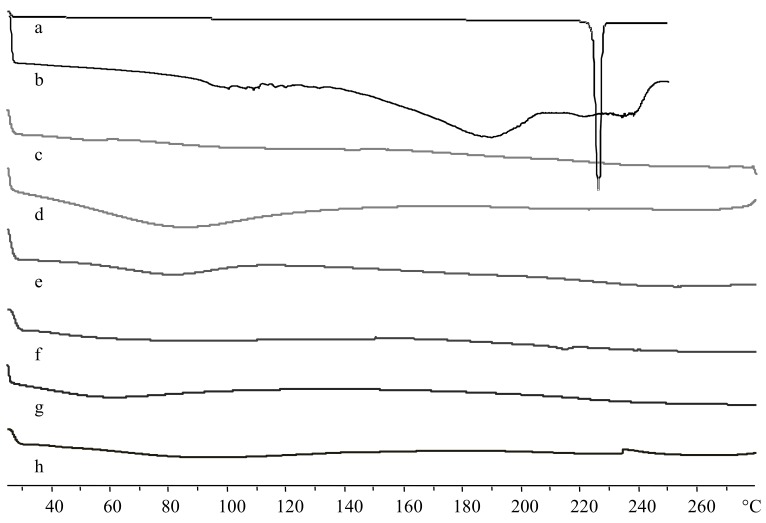
Differential Scanning Calorimetry (DSC) thermograms of TSPO ligands, carboxylated dextran derivatives and TSPO ligand conjugates: **1** (a), **4** (b), **6** (c), **9** (d), **7** (e), **8** (f), **10** (g), and **11** (h).

Figure 6 shows DSC thermograms of pure TSPO ligands **1** and **4**, C-Dex **6** and **9** and corresponding TSPO ligand–dextran conjugates—**7**, **8**, **10**, and **11**. The dextran derivatives and conjugates are amorphous and do not exhibit the endothermic crystalline melting peak of the pure TSPO ligand **1** at 226 °C indicating the absence of physically entrapped ligands. Therefore, the ligands are chemically attached to polymers **7** and **10**. Compound **4** shows a broad melting peak around 190 °C, followed by a degradation process, even though there was no evidence of this decomposition in the thermograms of polymers **8** and **11**.

### 2.5. In Vitro Stability Studies on TSPO LigandDextran Conjugates

**Figure 7:**
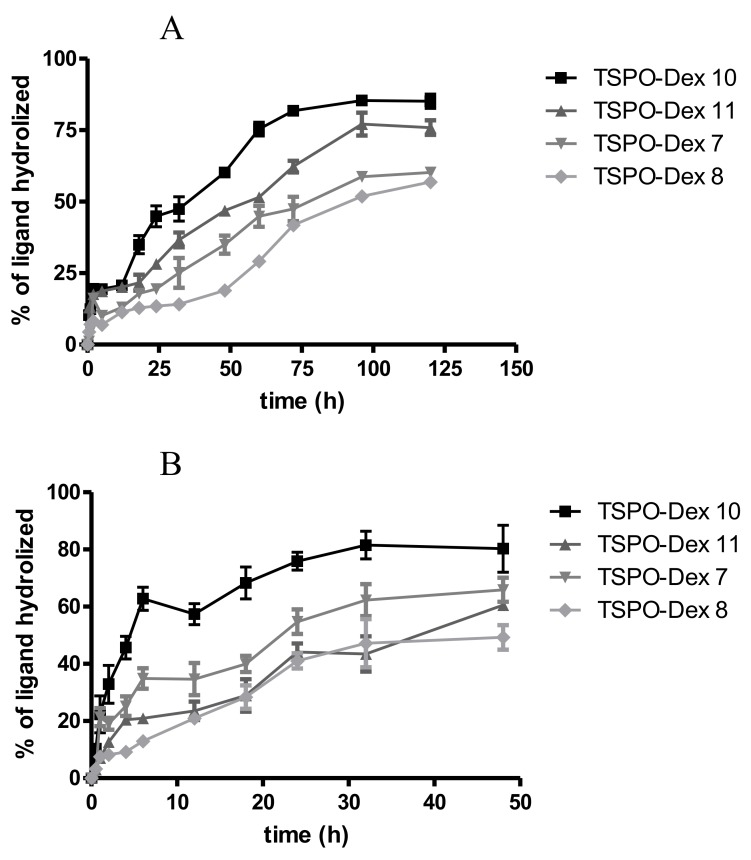
Hydrolysis profile of TSPO-DEX conjugates in (**A**) phosphate buffer (pH 7.4) at 37 ± 0.2 °C, and (**B**) diluted human serum at 37 ± 0.2 °C.

The hydrolysis rate of TSPO-Dex was evaluated in 0.05 M phosphate buffer at pH 7.4 (PBS), and in diluted human serum solution at 37 °C [23,24]. Figure 7 shows the release profile of TSPO ligands from conjugates in phosphate buffer (Figure 7A) and in human serum (Figure 7B). Conjugates characterized by ester binding (**7** and **10**) proved to be much less stable in PBS than the respective amide derivatives (**8** and **11**). In physiological medium, the hydrolysis of all conjugates is faster than that observed in phosphate buffer at pH 7.4. Indeed, TSPO ligand **1** in physiological solution was hydrolyzed from conjugate **10** within few hours, which was longer than hydrolyzation with conjugate **7**, probably due the presence of a double ester bound. The rank order of degradation after 24 h in human serum was as follows: 75.9 ± 5.4 (TSPO-Dex 10), 54.7 ± 7.4 (TSPO-Dex **11**), 44.1 ± 5.1 (TSPO-Dex **7**), 41.1 ± 4.6 (TSPO-Dex **8**) %. The degradation products of the TSPO-Dex in physiological medium were assessed after 24 h by LC-MS analysis in negative (ESI^−^) or positive (ESI^+^) mode. Mass spectra showed a peak at m/z 418, attributable to [M − H]^−^ of 1, for the conjugates **7** and **10** or a peak at m/z 442, attributable to [M + H]^+^ of **4** for the conjugates **8** and **11**, respectively. Moreover, other peaks pertaining to different hydrolysis reactions of conjugates were also detected and were attributable to the TSPO ligand moieties with spacers. In addition, TSPO ligand **3** was identified as a degradation product of conjugates **8** and **11**. 

### 2.6. Preparation and Characterization of the TSPO Ligand–Dextran NGs

**Figure 8:**
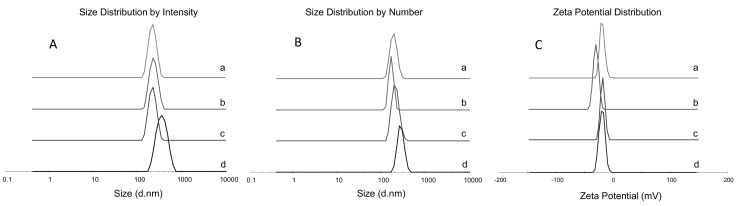
(**A**) Size distribution by intensity: TSPO-Dex NGs **7** (a); TSPO-Dex NGs **8** (b); TSPO-Dex NGs **10** (c) and TSPO-Dex NGs **11** (d). (**B**) Size distribution by number: TSPO-Dex NGs **7** (a); TSPO-Dex NGs **8** (b); TSPO-Dex NGs **10** (c) and TSPO-Dex NGs **11** (d). (**C**) Zeta Potential Distribution: TSPO-Dex NGs **7** (a); TSPO-Dex NGs 8 (b); TSPO-Dex NGs **10** (c) and TSPO-Dex NGs **11** (d).

TSPO-Dex conjugates have the ability to form physical hydrogels under physiological conditions. Therefore, TSPO-Dex NGs were prepared using the low temperature-induced PEG water-phase/dextran water-phase emulsion method, which is commonly used for engineering dextran nanoparticles [25] Briefly, TSPO-Dex and PEG aqueous solutions were mixed by magnetic stirring and the resulting clear solutions were immediately frozen and lyophilized. The lyophilized powder was then washed with CH_2_Cl_2_ to remove PEG. The NPs were collected as dry powder after drying of the residual organic phase. In this process, which is also known as the water-phase emulsion process, the low temperature during freezing induces the phase separation process between the internal aqueous phase of dextran and the external aqueous phase of PEG. Finally, dry NPs were rehydrated by resuspension of the powder in 0.05 M phosphate buffer at pH 7.4 and 25 °C to obtain the desired TSPO ligand decorated dextran NGs. Figure 8 and Table 3 summarize the yield, hydrodynamic size, polydispersity (PDI) and zeta potential of TSPO-Dex NGs prepared from TSPO-Dex conjugates 7, 8, 10 and 11. The redispersed dry nanoparticles in buffer showed a hydrodynamic diameter range of 280 to 420 nm, a number of diameters from 165 to 294 nm, and a PDI between 0.22 and 0.28, measured by dynamic light scattering (DLS). The zeta-average refers to the size distribution by intensity, whilst the number PDI depends on the number of particles. The values of PDI indicated a heterogeneous distribution of NGs, which was confirmed by electron microscopy (see below). The surface charges of the NGs were analyzed by measuring the zeta potential. In all cases, negative zeta potential values between −22 and −32 mV were detected due to the presence of free carboxylic groups (residues), suggesting good stability. (Figure 8C).

**Figure 9:**
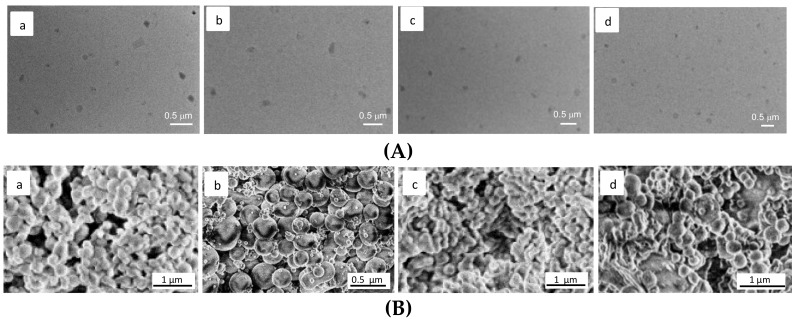
(**A**) (a–d of the top panel) scanning electron micrograph of TSPO-Dex NGs **7** (a); TSPO-Dex NGs **8** (b); TSPO-Dex NGs **10** (c) and TSPO-Dex NGs **11** (d). (**B**) (a–d of the bottom panel) transmission electron micrograph of TSPO-Dex NGs **7** (a); TSPO-Dex NGs **8** (b); TSPO-Dex NGs **10** (c) and TSPO-Dex NGs **11** (d).

The morphology investigation was carried out by means of transmission electron microscopy (TEM) and scanning electron microscopy (SEM). TEM micrographs of TSPO-Dex NGs **7**, **8**, **10** and **11** in Figure 9A highlighted that all NGs are characterized by a rather spherical shape and diameter values ranging from 90 to 190, 100 to 230, 120 to 250 and 150 to 400 nm, respectively. Therefore, the TEM investigation fundamentally confirmed the findings obtained from the DLS analysis, taking into account the shrinking of the NGs during the drying process performed before the TEM analysis. SEM measurements performed on TSPO ligand–dextran NGs and reported in Figure 9B provided diameter values comparable to those achieved by TEM analysis. Furthermore, in the case of TSPO-Dex NGs **7**, TSPO-Dex NGs **8**, and TSPO-Dex NGs **11**, SEM micrographs revealed the formation of tightly aggregated particles with a spherical shape and smooth surface. In contrast, NGs of TSPO-Dex **10** showed tightly aggregated particles with a rod-like shape and smooth surface that were characterized by a sponge network.

### 2.7. In Vitro Release Studies on TSPO Ligand–Dextran NGs

**Figure 10:**
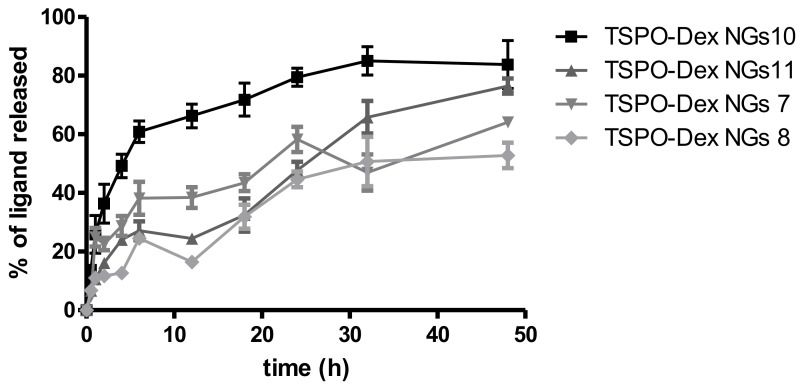
Release profile of TSPO-DEX nanogels (NGs) in diluted human serum at 37 ± 0.2 °C and pH 7.4.

The release of TSPO ligands from TSPO-Dex NGs was evaluated in diluted human serum solution at pH 7.4 and 37 °C, and the release profiles obtained are shown in Figure 10. As a whole, the experiments demonstrated that nanogels release the ligands with a profile similar to that seen for polymeric conjugates but with faster kinetics, probably due to their better hydration (as described later in the swelling studies) and the greater surface area of the nanogels exposed to the solvent. The rank order of percentage release after 24 and 48 h was as follows: 83.78 ± 14.2 and 79.4 ± 5.4 (TSPO-Dex NGs10), 76.4 ± 4.5 and 47.7 ± 5.1 (TSPO-Dex NGs 11), 64.1 ± 3.1 and 58.3 ± 7.4 (TSPO-Dex NGs 7), 52.8 ± 7.5 and 44.6 ± 4.6 (TSPO-Dex NGs 8) %.

### 2.8. In Vitro Swelling Studies

Studying the swelling of a polymer plays an important role in predicting its behavior as a hydrogel and its aptitude for use in the preparation of nanoparticulate systems (nanogels). C-Dex and TSPO-Dex have the ability to form physical hydrogels. The swelling degree of samples formulated as tablets was evaluated through the measurement of water absorbed by gravimetric analysis following Equation (2) and the results of this test are reported in Table 4. The swelling experiment was performed at 37 °C in physiological medium at pH 7.4 [26,27]. The data shows that the weights of all tablets increased during the experiment and became constant within two hours when they reach their equilibrium swelling state. After c.a. 240 min of incubation in a physiological environment, samples were extensively watted and converted into a jelly mass and further weighing was not possible. In particular, C-Dex **6** and **9** showed the highest swelling and reached equilibrium more quickly. All polymer and nanogel conjugates showed the same behavior with equilibrium times of around 90–120 min and 45–60 min, respectively. The rank order of swelling degree for polymers was as follows: 18.1 ± 2.40 (TSPO-Dex 7), 15.2 ± 2.4 (TSPO-Dex 8), 9.2 ± 3.0 (TSPO-Dex 11), 6.5 ± 2.1 (TSPO-Dex 10) %. Therefore, the swelling ability is probably due to the number of residual free carboxylic groups and therefore, the degree of conjugation. The nanogel tablets, prepared from freeze-dried nanoparticles, showed behavior similar to their starting polymers. In particular, they reached equilibrium faster than starting polymer, in around 45–60 min. Furthermore, the nanogels **7**–**8** showed a 25-fold increase in swelling degree compared to polymers **7**–**8**, and nanogels **10**–**11** showed a 20-fold increase in swelling degree compared to polymers **10**–**11**. This behavior is most probably due to the greater surface area of the nanogels exposed to the solvent, which makes the hydration of the polymeric network easier.

### 2.9. Rheological Study of Viscoelastic Properties

**Figure 11:**
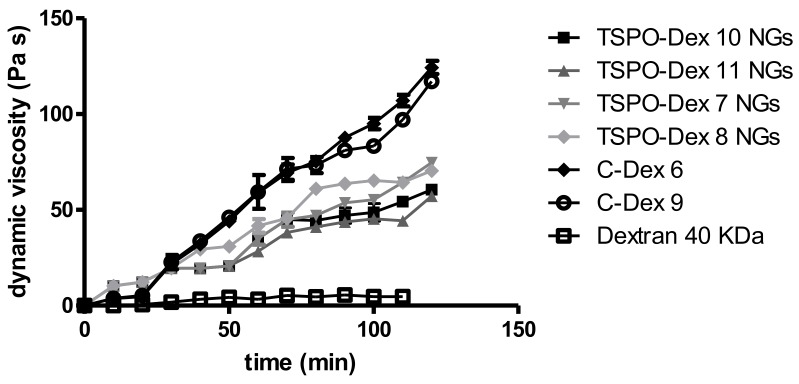
Rheological study of viscoelastic properties of TSPO-DEX NGs.

Hydrogels are characterized by viscoelastic properties that are slow due to their strength and gelling behaviour. Modulus G′ (the elastic modulus) is a measurement of elasticity while the loss modulus G″ represents the viscous components. This experiment was performed for as long as the gel network remained intact in the linear viscoelastic region. The data reported in Table 5 shows that the derivatization of dextran with chloroacetic acid (compound **6**) or succinic anhydride (compound **9**) led to a substantial increase in viscosity of up to 100-fold in phosphate buffer relative to dextran (40 kDa), which was used as a control. In fact, chloroacetic acid and succinic anhydride provided a free carboxylic group that could facilitate the establishment of hydrogen bonds as well as ionic interactions and gel strengthening of the polymer. Further, the conjugation with TSPO ligands led to a substantial decrease in dynamic viscosity to 70-fold of TSPO-Dex NGs relative to C-Dex. For polymers **6** and **9**, gelling was observed directly after preparation of the sample solutions. In addition, the carboxylated dextran derivatives showed instantaneous gelation at 25 °C, in comparison to TSPO-Dex for which sol–gel transition occurred after 2 h under the previously-stated conditions (Figure 11). The conjugation thus reduced the number of free carboxylic groups and consequently, reduced the gel strength, the G′ and G″ moduli and the dynamic viscosity. Finally, because all samples showed that G′ values predominate over the viscous modulus, G″, polymers **6** and **9**, and TSPO-Dex NGs can be considered to be solid-like materials, such as gels.

### 2.10. Cytotoxicity of the TSPO Ligand–Dextran NGs

The in vitro cytotoxicity of TSPO-Dex NGs **7**, **8**, **10** and **11** was studied by MTT conversion assay as the ability to interfere with cell survival in rat C6 glioma cell lines [28] C6 glioma cells represent an appropriate cellular model of study because their ability to express high concentrations of TSPO has been demonstrated [19]. Table 6 reports the half maximal inhibitory concentration (IC_50_) values calculated from dose–response curves for each sample. Dextran, which was used as a control at a concentration of 1 mg/mL, showed no toxicity, while TSPO ligands **1** and **4** exhibited IC_50_ values of 17.5 ± 0.7 and 19.2 ± 0.6 μM, respectively.

The rank order of IC_50_ values for TSPO ligand–dextran NGs formulations was as follows: 1.20 ± 0.07 (TSPO-Dex NGs 11), 1.85 ± 0.03 (TSPO-Dex NGs 10), 2.24 ± 0.06 (TSPO-Dex NGs 8) and 7.32 ± 0.15 (TSPO-Dex NGs 7) μM, respectively. The cytotoxicity of the different formulations appeared not to be dependent on the type of conjugated ligand but rather, on the conjugation degree and the rate of ligand release in physiological medium. Indeed, even though TSPO ligand **1** is slightly more cytotoxic than **4** on C6 cancer cells, polymer **7** (conjugated with TSPO ligand **1**) is less cytotoxic than the others, while polymers **10** (conjugated with TSPO ligand **1**) and **11** (conjugated with TSPO ligand **4**), which have a higher degree of conjugation and a higher ligand release speed after 24 h of incubation in physiological medium, are the most cytotoxic polymers. Therefore, the toxicity of these polymers seems to be linked precisely to the increased activation of the mitochondrial receptor of TSPO due to the greater degree of conjugation and of speed of drug release, regardless of the type of TSPO ligand used.

### 2.11. Uptake Studies of the TSPO Ligand–Dextran NGs

**Figure 12:**
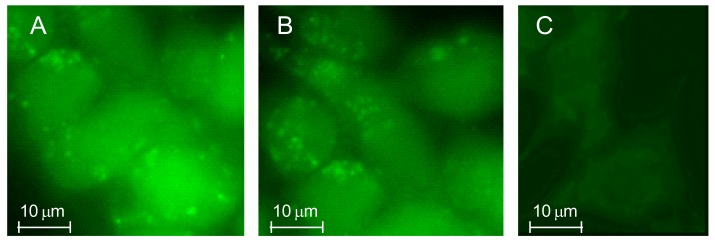
Rat C6 glioma cells were treated at 37 °C in a 5% CO_2_ atmosphere with 5 μM of TSPO–FITC–Dex NGs 10 (**A**) or TSPO-FITC-Dex NGs 11 (**B**) and the internalized fluorescence was imaged after 24 h of treatment. Images were obtained from 3 to 5 independent experiments and identical fields are presented. Untreated cells and cells treated with 5 μM FITC–Dex alone (after 24 h) were used as a control (**C**).

The internalization of TSPO ligand–dextran NGs into C6 glioma cells was studied in vitro by fluorescence microscopy. For this purpose, fluorescent TSPO ligand–dextran NGs were prepared using FITC–Dextran and TSPO-Dex **10** or **11** (TSPO-FITC-Dex NGs) in order to explore NGs decorated with TSPO ligands **1** and **4**. Cells incubated with 5 μM of TSPO–FITC–Dex NGs (concentration refers to FITC) showed a widely diffuse green fluorescence in the cytosol after 24 h, as reported in Figure 12. The fluorescence in cells was exclusively due to the cellular uptake of TSPO–FITC–Dex NGs because no green fluorescence was imaged when cells were incubated in the presence of 5 μM FITC–Dextran.

## 3. Experimental Section

### 3.1. Materials and Methods

Dextran, **5** (from *Leuconostoc* spp., average mol wt 40,000 Da, M_r_), FITC–Dextran (average mol wt 40,000 Da, FITC: Glucose = 1:250), chloroacetic acid, succinic anhydride, LiCl, *N*-(3-dimethylaminopropyl)-*N*′-ethylcarbodiimide hydrochloride (EDC), 1,1′-carbonyldiimidazole (CDI), 1-hydroxybenzotriazole hydrate (HOBt), triethylamine (TEA), anhydrous DMF and anhydrous pyridine were purchased from Sigma-Aldrich (Milan, Italy). All other chemicals were of analytical grade and were used without further purification. The synthetic procedures to prepare TSPO ligands, *N*,*N*-di-*n*-propyl-[2-(6,8-dichloro-2-(4-hydroxyphenyl)imidazo[1,2-a]pyridin-3-yl)]-acetamide **1** and *N*,*N*-di-*n*-propyl-[2-(8-(2-aminoacetamido)-2-(4-chlorophenyl)imidazo[1,2-a]pyridin-3 yl)]-acetamide **4**, have been previously reported elsewhere [19,29].

^1^H-NMR spectra were recorded on a Varian Mercury 300 MHz instrument. Chemical shifts were expressed in ppm and referenced by solvent using the residual protic peak (DMSO-d6, 2.50 ppm and D_2_O, 4.80 ppm) [30]. FT-IR spectra were sampled in KBr pellets and were recorded on a Perkin Elmer 1600 FT-IR spectrophotometer (Spectrum One). UV-VIS spectroscopy was accomplished using a Perkin-Elmer Spectrometer Lamba Bio20 [31]. Elemental analyses were carried out with an Eurovector EA 3000 CHN elemental analyzer. Freeze-drying was performed using Christ Alpha 1-4 LSC, and all samples were dried for 24 h under reduced pressure (0.016 mbar) at −60 °C. DSC thermograms were acquired by a Mettler Toledo DSC 822e (Stare 202 System) equipped with a thermal analysis automatic program and using indium as an internal standard. HPLC analyses were performed with a Waters Associates (Milford, MA, USA) Model 1515 HPLC isocratic pump with a Symmetry C18 (4.6 × 150 mm, 5 μm) column, a Waters 2487 UV-Vis detector and Breeze Software to analyse the chromatographic data. TSPO-ligands **1** and **4** were quantified using a mixture of water/methanol in a ratio of 30:70 (*v*/*v*) as the eluent at a flow rate of 0.8 mL/min and a wavelength of 254 nm. The data were processed using Microsoft Excel 2010 or GraphPad Software [32].

### 3.2. Synthesis of TSPO Ligand–Dextran Conjugates

The carboxylated dextran derivatives **6** and **9** and the TSPO-Dex conjugates **7**, **8**, **10** and **11** were prepared according to the synthetic procedures reported in Figure 2 and Figure 3.

#### 3.2.1. Synthesis of the Carboxylated Dextran Derivatives **6** and **9**

Chloroacetic acid (6.0 g, 64 mmol) was added to a stirred solution of **5** (2.0 g, 50 µmol) in 6N NaOH (20 mL), and the resulting solution was stirred at 70 °C for 4 h. After this time, the reaction mixture was cooled at room temperature and the pH was corrected to pH 5.0 with acetic acid. The resulting mixture was concentrated under vacuum and then dialyzed with membrane tubing (Spectra/Por^®^ 10,000 MWCO, RC) against deionized water. To achieve a complete purification of samples, the water was changed three to five times per day for three days. The purified dispersion was freeze-dried, and the obtained compound (compound **6**) was stored at 4 °C until further use. Compound **9** was prepared through a reaction of **5** (2.0 g, 50 µmol) with succinic anhydride (3.7 g, 37 mmol) in the presence of LiCl (0.4 g, 9.5 mmol) and anhydrous pyridine (1.5 mL) in anhydrous DMF (20 mL) at 80 °C, in a nitrogen atmosphere. The reaction mixture was stirred overnight and then concentrated under vacuum and transferred into dialysis membrane tubing. The successive purification by dialysis and freeze-drying was carried out as described above to give compound **9**.

Compound **6**: FTIR (KBr): 1732 cm^−1^ (υ_C=O_); ^1^H-NMR (D_2_O) δ: 3.2–4.2 (m, H_2_–H_6_, glucose ring protons), 4.81 (m, H_1_, glucose ring proton), 4.99 (m, -O–CH_2_–COO).

Compound **9**: FTIR (KBr): 1736 (υ_C=O_, COO–glucose), 1731 cm^−1^ (υ_C=O_, COOH); ^1^H-NMR (D_2_O) δ: 2.33 (m, -CO–CH_2_–CH_2_–COO), 2.51 (m, -CO–CH_2_–CH_2_–COO), 3.2–4.2 (m, H_2_–H_6_, glucose ring protons), 4.81 (m, H_1_, glucose ring proton).

#### 3.2.2. Synthesis of the TSPO Ligand–Dextran Conjugates **7** and **10**

EDC (200 mg, 1.0 mmol) and HOBt (143 mg, 1.2 mmol) were added to a stirred mixture of 6 (0.5 g, 5.5 μmol) in anhydrous DMF (20 mL) maintained at room temperature and under a nitrogen atmosphere. After 10 min, the mixture was treated with a solution of compound 1 (170 mg, 0.40 mmol) and TEA (100 μL) in anhydrous DMF (5 mL) and stirring was prolonged for 12 h and then concentrated under vacuum and transferred into dialysis membrane tubing. The successive purification by dialysis and freeze-drying was carried out as described above to give compound 7. Dextran conjugate 10 was prepared following the same procedure using compound 9 (0.5 g, 4.1 μmol).

Compound **7**: IR (KBr): 1745 cm^−1^ (υ_C=O_, COO), 1601 cm^−1^ (υ_C=O_, CON); ^1^H-NMR (DMSO-d_6_) δ: 0.7–0.9 (m, 6H, CH_3_), 1.0–1.8 (m, 4H, CH_2_), 3.0–4.8 (m, CH_2_NCO and CH_2_CON overlapping with H_1_–H_6_ of the glucose ring protons), 5.0 (m, -O–CH_2_–COO), 6.82 (d, *J* = 8.5 Hz, Aromatic H), 7.1–7.3 (m, Aromatic H), 7.41 (d, *J* = 8.5 Hz, Aromatic H), 7.5–7.7 (m, Aromatic H), 8.5–8.7 (m, Aromatic H).

Compound **10**: IR (KBr): 1744 cm^−1^ (υ_C=O_, COO), 1610 cm^−1^ (υ_C=O_, CON); ^1^H-NMR (DMSO-*d*_6_) δ: 0.7–0.9 (m, 6H, CH_3_), 1.0–1.8 (m, 4H, CH_2_), 2.2–2.6 (m, -CO–CH_2_–CH_2_–COO), 3.0–5.2 (m, CH_2_NCO and CH_2_CON overlapping with H_1_–H_6_ of the glucose ring protons), 6.82 (d, *J* = 8.5 Hz, Aromatic H), 7.1–7.3 (m, Aromatic H), 7.41 (d, *J* = 8.5 Hz, Aromatic H), 7.5–7.7 (m, Aromatic H), 8.5–8.7 (m, Aromatic H).

#### 3.2.3. Synthesis of the TSPO Ligand–Dextran Conjugates **8** and **11**

CDI (200 mg, 1.2 mmol) was added to a stirred mixture of 6 (0.5 g, 5.5 µmol) in anhydrous DMF (20 mL) maintained at room temperature and under nitrogen atmosphere. After 30 min, the mixture was treated with a solution of compound **4** (170 mg, 0.38 mmol) and TEA (100 μL) in anhydrous DMF (5 mL) and stirring was prolonged for 12 h and then concentrated under vacuum and transferred into dialysis membrane tubing. Successive purification by dialysis and freeze-drying was carried out as described above to give compound **8**. Dextran conjugate **11** was prepared following the same procedure using compound **9** (0.5 g, 4.1 µmol).

Compound **8**: IR (KBr): 1600 cm^−1^ (υ_C=O_, CON); ^1^H-NMR (DMSO-*d*_6_) δ: 0.7–0.9 (m, 6H, CH_3_), 1.0–1.8 (m, 4H, CH_2_), 3.0–4.8 (m, CH_2_NCO and CH_2_CON overlapping with H_1_–H_6_ of the glucose ring protons), 5.0 (m, -O–CH_2_–COO), 6.82 (d, *J* = 8.5 Hz, Aromatic H), 7.1–7.3 (m, Aromatic H), 7.46 (d, *J* = 8.5 Hz, Aromatic H), 7.69 (d, *J* = 8.5 Hz, Aromatic H), 8.2–8.4 (m, Aromatic H).

Compound **11**: IR (KBr): 1740 cm^−1^ (υ_C=O_, COO), 1610 cm^−1^ (υ_C=O_, CON); ^1^H-NMR (DMSO-d_6_) δ: 0.7–0.9 (m, 6H, CH_3_), 1.0–1.8 (m, 4H, CH_2_), 2.4–2.8 (m, -CO–CH_2_–CH_2_–COO), 3.0–5.2 (m, CH_2_NCO and CH_2_CON overlapping with H_1_–H_6_ of the glucose ring protons), 6.82 (d, *J* = 8.5 Hz, Aromatic H), 7.1–7.3 (m, Aromatic H), 7.46 (d, *J* = 8.5 Hz, Aromatic H), 7.69 (d, *J* = 8.5 Hz, Aromatic H), 8.2–8.4 (m, Aromatic H).

### 3.3. Size Exclusion Chromatography Analysis

The average molecular weights and molecular weight distributions of the TSPO-Dex were evaluated by size exclusion chromatography (SEC) using a Waters Associates (Milford, MA, USA) Model 1515 HPLC isocratic pump and with Waters Breeze software to process chromatographic data. HPLC was equipped with an UltraHydrogel TM 500 column (7.8 × 300 mm, 5 µm) and a Waters 2414 differential RID detector. As a mobile phase, 0.25 M of a phosphate buffer solution with pH 7.2 was used at 30 °C with a flow rate of 0.8 mL/min. Test solutions were prepared in phosphate buffer solution at pH 7.2 at a concentration of 3 mg/mL, and 20 µL samples were injected onto the column. Dextran standards (Mp = 10,000–1,100,000 Da, Sigma-Aldrich) were used for calibration.

### 3.4. Determination of the Substitution Degree of Carboxylic Groups in the Carboxylated Dextran Derivatives

The carboxylated dextran derivatives **6** and **9**, and the TSPO-Dex conjugates **7**, **8**, **10** and **11** were prepared according to the synthetic procedures reported in Figure 2 and Figure 3. The number of total carboxylic groups of C-Dex **6** and **9** was estimated by ^1^H-NMR spectroscopy, while the acid number (*n*; mg NaOH/g C-Dex) was determined by acid–base titration. Briefly, derivatives were dissolved in 0.01 M NaCl (5 mg/mL), and the solutions were titrated by the stepwise addition of 0.01 M NaOH to obtain the titration profile using a pH meter (SevenExcellence, Mettler Toledo, Greifensee, Switzerland) equipped with a combination of polymer electrodes of varied pH (InLab Expert Pro, Mettler Toledo, WA, USA).

### 3.5. Determination of TSPO Ligand Contents on TSPO Ligand–Dextran Conjugates

The conjugation degree (CD %) of the TSPO-Dex (i.e., the percentage of TSPO ligand linked to the polymeric carrier) was estimated by UV spectroscopy and confirmed using molecular weights. The molecular weights were calculated by SEC analysis using the following Equation:

(1) $$CD\left(\%\right)=\frac{Mn\left(TSPO-Dex\right)-Mn\left(C-Dex\right)}{\left[MW\left(TSPO-ligand\right)-18\right]n}\times 100$$

where *M_n_* (TSPO-Dex) is the number average molecular weight of TSPO ligand–dextran conjugates **7**, **8**, **10** and **11**, *M_n_* (C-Dex) is the number average molecular weight of carboxylated dextran derivatives **6** and **9** and *M_W_* (TSPO ligand) is the molecular weight of TSPOligand **1** or **4**. The value 18 is the molecular weight of a molecule of water that is eliminated in the conjugation reaction, and *n* = 154.9 is the degree of polymerization.

### 3.6. Differential Scanning Calorimetry (DSC) Studies

DSC analyses of dextran, carboxylated dextran, TSPO ligands and TSPO-Dex were done to investigate the physicochemical behavior of NG polymers. Samples (5 mg) were placed in an aluminum pin and heated from 25 to 280 °C at a rate of 5 °C min^−1^ under a nitrogen flow of 50 cm^3^ min^−1^ [33].

### 3.7. Stability Studies of the TSPO Ligand–Dextran Conjugates in Phosphate Buffer Solution

The chemical hydrolysis of TSPO-Dex was studied at pH 7.4 in 50 mM phosphate buffered saline (PBS) at 37 °C. The experiments were carried out by pouring 5 mL of a TSPO-Dex solution at a concentration of 1 mg/mL into a dialysis bag (Spectra/Por^®^, MWCO = 3.5 kDa). The dialysis bag was placed into 10 mL of the buffer, preheated at 37 °C, and the resulting mixture was maintained in a shaker water bath at constant temperature of 37 ± 0.2 °C. Aliquots of 500 μL of external medium were removed at suitable time intervals, filtered through cellulose acetate membranes (0.2 µm, 13 mm, Teknokroma Analitica, Barcelona, Spain), and 20 µL of filtrate was analyzed by HPLC. After each draw, an equal volume of fresh external medium was replaced to keep the sink conditions. The TSPO ligands were quantified by measuring the peak area in relation to standard solutions of the ligands analyzed by HPLC under identical conditions.

### 3.8. Stability Studies of the TSPO Ligand–Dextran Conjugates in Human Serum Solution

The TSPO-Dex conjugates were investigated in regard to their susceptibility to hydrolysis at 37 °C and pH 7.4 in a solution of dilute rat serum with 0.05 M of PBS, using NaCl as an isotonic agent. The studies were carried out on 1% sample solutions obtained by mixing 0.1 mL of 1 mg/mL stock solution of TSPO-Dex in DMSO with 1 mL of preheated 50% *v*/*v* rat serum solution which had been kept in a shaker water bath at constant temperature of 37 °C (± 0.2 °C) during the experiment. Then, aliquots of 100 μL of sample solution were removed at suitable time intervals, mixed with 0.5 mL of cold acetonitrile and filtered through cellulose acetate membranes (0.2 µm, 13 mm Φ, Teknokroma Analitica, SA, Spain), and 20 µL of each filtrate was analyzed by HPLC. The TSPO ligands were quantified by measuring the peak area in relation to standard solutions of the ligands analyzed by HPLC under identical conditions. The measurements were done in triplicate.

### 3.9. Preparation of the TSPO Ligand–Dextran NGs

The TSPO ligand–dextran NGs (TSPO-Dex NGs) were prepared as follows: 5 mL of a TSPO-Dex solution (5% *w*/*w*) was gently mixed with 5 mL of PEG solution (25% *w*/*w*), followed by stirring for 60 s to obtain a clear solution. After this, the solution was immediately frozen using liquid nitrogen and then freeze-dried for 24 h (vacuum level below 0.16 mbar). The freeze-dried powder was suspended in 1 mL of CH_2_Cl_2_ and washed three times with more 4 mL of CH_2_Cl_2_ to extract the PEG and then subsequently centrifuged at 13,000 rpm for 15 min to remove the continuous phase containing the PEG. Finally, after the residual organic phase was evaporated under vacuum, dry NGs were obtained.

### 3.10. Physicochemical Characterization of TSPO Ligand–Dextran NGs

#### 3.10.1. Average Hydrodynamic Diameters, Polydispersity and Zeta Potential

The average hydrodynamic diameter (z-average), mean hydrodynamic diameter number (number PSD), size distribution (polydispersity index, PDI) and zeta potential (ζ) of the TSPO-Dex NGs were measured by dynamic light scattering (DLS) using a Zetasizer Nano ZS (Malvern Instruments Ltd., Worcestershire, UK) after suitable dilution of clear suspensions in demineralized water (0.1 mg/mL). The zeta potential of TSPO ligand–dextran NGs was determined by laser Doppler velocimetry with the same instrument and after dilution with 1 mM KCl at the concentration of the dispersion (0.1 mg/mL).

#### 3.10.2. Transmission Electron Microscopy Investigation

A Jeol JEM-1011 microscope, working at an accelerating voltage of 100 kV, was used to perform the morphological characterization of TSPO-Dex NGs by means of Transmission Electron Microscopy (TEM). TEM micrographs were acquired by an Olympus Quemesa Camera (11 Mpx).

#### 3.10.3. Field Emission Scanning Electron Microscopy Investigation

Field emission scanning electron microscopy (FE-SEM) measurements were carried out using a Zeiss Sigma FE-SEM, with a working electron high tension (EHT) of 0.5–20 kV, equipped with an in-lens secondary electron detector [34].

### 3.11. Release Studies in Human Serum Solution of the TSPO-Ligand-Dextran Nanogels

The TSPO-Dex NGs were investigated at 37 °C and pH 7.4 in a solution of dilute rat serum with 0.05 M of PBS, using NaCl as an isotonic agent. The studies were carried as previously described in Section 3.8.

### 3.12. Swelling Studies

All samples were dried under vacuum for 24 h before the swelling studies were carried out. Tablets of the TSPO ligand–dextran polymers (30 mg) or TSPO-Dex NGs (10 mg) were obtained followed a previously reported procedure [35]. The tablets obtained were put into a stainless steel grid and immersed in 10 mL of phosphate buffered saline solution (PBS, pH 7.4) at 37 ± 0.5 °C using a tube with a screw cup. The swelling degree of each sample was evaluated through the measurement of water absorbed by gravimetric analysis of the swollen tablets. For this purpose, the steel grid with the tablets was taken out at scheduled time and up two hours (final measurements were taken until the equilibrium was reached), and after removing excess water with a sheet of absorbent paper, the amount of absorbed water was calculated with Equation (2):

(2) $$Swelling\left(\%\right)=\frac{W_{t}\left(Tablets\right)-W_{0}\left(Tablets\right)}{W_{0}\left(Tablets\right)}\times \;100$$

where *W_t_* (Tablets) is the tablet weight at each time point and *W*_0_ (Tablets) the initial tablet weight, respectively.

### 3.13. Rheological Study of Viscoelastic Properties

The rheological study of the viscoelastic properties of the polymers and NGs was performed according to a method previously reported using a thermostatically-controlled cone-plate combination rheometer. (Haake Mars Rheometer, 379-0200, Thermo Electron GmbH, Karlsruhe, Germany; Rotor: Ci35 Ti, D = 35 mm) [36]. Rheological behaviour was determined by hydrating unmodified derivatives, conjugate polymers and NGs in 0.25 M phosphate buffer at pH 7.4 to reach a final concentration of 5% (*m*/*v*). After complete hydration, the obtained polymer solutions were allowed to equilibrate for 20 min. The dynamic oscillatory test was carry out at a frequency of 1 Hz in the straightforward viscoelasticity region. The shear stress rate was executed at 25 ± 1 °C and a range of 0.5–1 Pa was accomplished using a gap of 0.5 mm between the two plates. The elastic modulus (G′), the viscous modulus (G″) and the dynamic viscosity (η) were collected. Each measurement was performed in quintuplicate.

### 3.14. Cell Cultures and Cell Viability Analysis by MTT Assay

Rat C6 glioma cells were grown at 37 °C in a humidified 5% CO_2_ atmosphere in DMEM (Dulbecco’s modified Eagle’s medium, EuroClone, Milan, Italy), supplemented with 10% FBS (Fetal Bovine Serum, EuroClone, Italy), 2 mM l-glutamine, penicillin (100 U/mL) and streptomycin (100 µg/mL). Cells were fed every day and seeded at a density of ~5000 cells/well in 96-well plates for viability assays, or at a density of ~100,000 cells/well in 6-well plates for fluorescence microscopy analysis. 

Cells were incubated with different concentrations of TSPO ligands (10–100 μM) and TSPO-Dex NGs (0.1–10 mg/mL) for 24 h. Cell viability was evaluated by quantitative colorimetric tetrazolium salt reduction (MTT) assay, as previously described [10], and the absorbance of an individual well was measured with a microplate reader (Wallac Victor3, 1420 Multilabel Counter, Perkin-Elmer, Waltham, MA, USA).

### 3.15. Fluorescence Microscopy

The uptake of NGs into rat C6 glioma cells was determined by fluorescence measurements performed through an inverted Zeiss Axiovert 200 microscope (Zeiss, Milano, Italy), equipped with a 63 × 1.4 oil objective, as previously described [37]. NGs were tested at a concentration of 5 μM (concentration refers to FITC) and the internalized fluorescence was imaged after 24 h after the treatment. FITC-labelled NGs were evaluated by fitting excitation and emission wavelengths with a Zeiss 10 filter set at BP 450–490 nm (Beamsplitter, D.M. FT 510 nm) for excitation and 540BP25 nm for emission [20]. The acquisitions of fluorescence images were carried out with a CoolSNAP HQ CCD camera (Roper Scientific, Trenton, NJ, USA) and then processed by Metamorph/Metafluor software (U.I.C., West Chester, PA, USA).

In each experiment, 20–30 cells were selected and measurements were repeated 5 times using cells from independent cultures.

### 3.16. Statistical Analysis

All data are presented as means ± S.D. The statistical significance of differences was calculated by one-way analysis of variance (ANOVA) followed by the Bonferroni’s Multiple Comparison Test (*p* < 0.05 was considered statistically significant) (GraphPad Prism version 5 for Windows, GraphPad Software, San Diego, CA, USA).

## 4. Conclusions

NGs are ideal candidates for the delivery of biopharmaceuticals in cancer cells because they can be chemically modified with selected ligands for cellular or subcellular drug targeting. In the present work, TSPO ligand–dextran conjugate NGs, based on biodegradable dextran, were used as a carrier for the efficient delivery of pro-apoptotic TSPO ligands into cancer cells overexpressing TSPO. 

## Figures and Tables

**Table 1:** Average molecular weights and the polydispersity index (PDI) determined by Size Exclusion Chromatography (SEC) and elemental analysis of dextran **5**, carboxylated dextran derivatives **6** and **9**, and TSPO ligand conjugates **7**, **8**, **10** and **11**.

Compound	*M_n_*^a^	*M_w_*^b^	*M_p_*^c^	PDI	Elemental Analysis					
					Calculated ^d^			Found		
	(kDa ± SD)			(*M_w_*/*M_n_*)	%C	%H	%N	%C	%H	%N
**5**	25.1 ± 0.5	42.9 ± 0.5	27.7 ± 0.5	1.71	44.44	6.17	-	44.19	6.60	-
**6**	56.1 ± 0.5	105.5 ± 2.5	64.4 ± 0.5	1.88	46.22	5.94	-	44.27	5.99	-
**7**	73.3 ± 2.5	128.4 ± 1.8	82.2 ± 0.9	1.75	48.69	5.86	3.83	48.08	5.90	3.59
**8**	79.9 ± 0.4	155.8 ± 2.2	87.7 ± 1.5	1.95	49.27	5.44	3.68	48.52	5.56	3.10
**9**	62.3 ± 0.5	117.7 ± 2.0	77.2 ± 0.7	1.89	44.87	5.91	-	44.84	5.93	-
**10**	85.4 ± 1.2	161.4 ± 5.5	90.8 ± 2.0	1.89	51.38	5.64	3.77	50.83	5.68	3.47
**11**	80.7 ± 0.5	162.2 ± 8.2	89.0 ± 1.0	2.01	52.66	4.95	5.91	52.04	5.04	5.47

^a^ Number average (*M_n_*) molecular weight; ^b^ weight average (*M_w_*) molecular weight; ^c^ molar mass at the peak maximum molecular weight (*M_p_*); ^d^ Calculated on the basis of the TSPO ligand conjugation degree (see Table 2).

**Table 2:** Yield, acid number (*n*) and carboxylic group substitution degree of the carboxylated dextran derivatives **6** and **9** and conjugation degrees of TSPO ligand conjugates **7**, **8**, **10** and **11**.

Compound	Yield %	pH Analysis	UV Analysis			^1^H-NMR Analysis		Elemental Analysis	SEC Analysis
		*n*	E^1% c^	λ_max_ (nm)	TSPO Ligand Conjugation Degree	Carboxylic Group Substitution Degree	Residual Free Carboxylic Groups	TSPO Ligand Conjugation Degree	TSPO Ligand Conjugation Degree
	(*w*/*w*) ^a^	(mg/g) ^b^			(g/g) ^e^	(mol/mol) ^f^		(g/g) ^e^	(mol/mol) ^g^
**1**			62.8 ^d^	253					
**4**			76.5 ^d^	254					
**6**	82	55.7				0.51			
**7**	68		23.2	250	0.37		0.23	0.31	0.28
**8**	51		25.2	254	0.33		0.26	0.28	0.25
**9**	80	54.2				0.55			
**10**	71		36.4	251	0.58		0.18	0.55	0.37
**11**	54		43.6	254	0.57		0.20	0.52	0.35

^a^ Based on the percentage by weight of the polymer synthesized and the starting dextran; ^b^ calculated as mg of NaOH per g of polymer; ^c^ determined by UV analysis in H_2_O at wavelength 254 nm; ^d^ determined by UV analysis in MeOH:H_2_O, at a ratio of 2:1, at a wavelength of 254 nm; ^e^ calculated as g of TSPO ligand/g of polymer; ^f^ calculated as number of moles of carboxylic groups of C-Dex per mole of glucose; ^g^ calculated with Equation (1).

**Table 3:** Physicochemical characteristics of TSPO-Dex NGs.

NGs	Size *d*_mean_ (nm)		PDI ^c^	ζ (mV) ^d^
	z-Average ^a^	Number PSD ^b^		
7	320.8 ± 14.13	180.9 ± 1.8	0.24 ± 0.11	−28.1 ± 2.1
8	280.8 ± 12.45	165.3 ± 2.8	0.22 ± 0.10	−32.0 ± 5.2
10	391.2 ± 16.99	220.5 ± 5.7	0.27 ± 0.06	−22.1 ± 2.4
11	420.4 ± 34.11	294.4 ± 6.3	0.28 ± 0.09	−25.5 ± 3.3

^a^ Average hydrodynamic diameter; ^b^ Average number of hydrodynamic diameters; ^c^ PDI = polydispersity index; ^d^ ζ = zeta potential; means ± SD are reported, *n* = 3.

**Table 4:** Swelling degree of C-Dex, TSPO-Dex conjugates and TSPO-Dex NGs after 2 h.

Tablet		Swelling PBS pH 7.4			
Polymer	Nanogels	Polymer		Nanogels	
		t, min	(*w*/*w*, %)	t, min	(*w*/*w*, %)
TSPO-Dex 7	TSPO-Dex NGs 7	120	18.1 ± 2.0	45	22.6 ± 2.5
TSPO-Dex **8**	TSPO-Dex NGs **8**	120	15.2 ± 2.4	45	19.0 ± 1.9
TSPO-Dex **10**	TSPO-Dex NGs **10**	90	6.5 ± 2.1	60	7.8 ± 0.7
TSPO-Dex **11**	TSPO-Dex NGs **11**	90	9.2 ± 3.0	60	11.1 ± 1.0
C-Dex **6**	-	20	31.5 ± 4.6	-	-
C-Dex **9**	-	20	27.4 ± 2.5	-	-

Measurements in the swelling study were performed in triplicate (means ± SD are shown). The values reported were taken at equilibrium (when three consecutive determinations gave the same weight).

**Table 5:** Rheological results of TSPO-Dex NGs.

Nanogels	G′ (Pa)	G″ (Pa)	η (Pa s)
**6**	1320 ± 56.5	220 ± 16.0	125.1 ± 2.5
**9**	1191 ± 48.3	182 ± 15.5	117.2 ± 9.4
TSPO-Dex NGs **7**	870 ± 66.5	85 ± 6.6	75.1 ± 2.5
TSPO-Dex NGs **8**	801 ± 28.8	82 ± 8.5	70.2 ± 6.5
TSPO-Dex NGs **10**	720 ± 33.5	74 ± 6.7	63.5 ± 1.9
TSPO-Dex NGs **11**	683 ± 37.2	68 ± 5.5	57.4 ± 2.6

G′ is the elastic modulus, G″ is the viscous modulus and η is the dynamic viscosity.

**Table 6:** Cytotoxicity against C6 Glioma cells of TSPO-Dex NGs.

Sample	IC_50_ (µM) ^a^
**1**	17.5 ± 0.7
**4**	19.2 ± 0.6
TSPO-Dex NGs **7**	7.32 ± 0.15
TSPO-Dex NGs **8**	2.24 ± 0.06
TSPO-Dex NGs **10**	1.85 ± 0.03
TSPO-Dex NGs **11**	1.20 ± 0.07

^a^ The half maximal inhibitory concentration (IC_50_) in terms of TSPO ligand concentrations. Values are means ±  SD of three experiments performed in triplicate.

## Abbreviations

The following abbreviations are used in this manuscript:

CBR	Central-type benzodiazepine receptor
C-Dex	Carboxylated dextran derivatives
CDI	1,1′-carbonyldiimidazole
DSC	Differential scanning calorimetry
EDC	*N*-(3-dimethylaminopropyl)-*N*′-ethylcarbodiimide hydrochloride
HOBt	1-hydroxybenzotriazole hydrate
MPTP	Mitochondrial permeability transitional pore
NGs	Nanogels
NPs	Nanoparticles
PBR	Peripheral-type benzodiazepine receptor
PBS	Phosphate buffered saline
PLGA	poly(d,l-lactic-*co*-glycolic acid)
SEM	Scanning electron microscopy
TEA	Triethylamine
TEER	Transendothelial electrical resistance
TEM	Transmission electron microscopy
TSPO	Translocator protein 18 kDa
TSPO-Dex	TSPO ligand–dextran conjugates
